# Rapid detection of avian leukemia virus using CRISPR/Cas13a based lateral flow dipstick

**DOI:** 10.3389/fvets.2024.1424238

**Published:** 2024-08-16

**Authors:** Jing Li, Zichuang Zhang, Zongshu Zhang, Xi Chen, Chunguang Wang, Xianghe Zhai, Tie Zhang

**Affiliations:** ^1^College of Veterinary Medicine, Hebei Agricultural University, Baoding, China; ^2^Institute of Special Animal and Plant Sciences, Chinese Academy of Agricultural Sciences, Changchun, China

**Keywords:** avian leukemia virus, recombinase-aided amplification, CRISPR/Cas13a, lateral flow dipstick, rapid detection

## Abstract

Avian leukemia virus (ALV) is one of the main pathogens of poultry tumor diseases, and has caused significant economic losses to the poultry industry since its discovery. Therefore, establishing a rapid detection method is essential to effectively prevent and control the spread of ALV. In this study, specific CRISPR RNA (crRNA) and recombinase-aided amplification (RAA) primers with T7 promoter were designed based on the relatively conserved sequence of avian leukemia virus. When crRNA recognized the target sequence, Cas13a protein was activated to cut the reporting probes, and then the detection results were read by using lateral flow dipstick (LFD). The RAA-CRISPR/Cas13a-LFD reaction system was constructed. The RAA amplification time, Cas13a protein concentration, crRNA concentration and CRISPR reaction time were optimized to evaluate the specificity, sensitivity and reproducibility of the system. Finally, RAA-CRISPR/Cas13a-LFD method was compared with Polymerase chain reaction (PCR)-Agarose electrophoresis method and qPCR method in the detection of clinical samples, and the reliability of RAA-CRISPR/Cas13a-LFD method was evaluated. The results showed that the RAA-CRISPR/Cas13a-LFD method could effectively amplify the target gene at 37°C for 40 min, and the test results could be determined by LFD visual observation. The method had good specificity and no cross-reaction with Marek’s disease virus (MDV), Fowl adenovirus (FAdV), Infectious bursal disease virus (IBDV), Newcastle disease virus (NDV), Infectious laryngotracheitis virus (ILTV), and Infectious bronchitis virus (IBV). The minimum detection limit of the method was 10^0^ copies/μL, and it had good repeatability and stability. The coincidence rate of clinical detection reached 97.69% and 99.23%. In summary, this study established a simple, efficient, accurate and visualized ALV detection method, which can be used for the prevention and rapid clinical diagnosis of avian leukosis (AL).

## Introduction

Avian leukosis (AL) is an immunosuppressive disease caused by avian leukemia virus (ALV) infection (1). The main manifestations of the disease are multi-organ tumor, immune suppression, and easy to secondary infections from other pathogens. It leads to direct economic loss after production performance decline, which seriously affects the healthy development of poultry industry (2). Among the known 11 ALV subgroups, the most infected chickens in China were subgroup J, followed by subgroup A and subgroup B (3). ALV is a common naturally occurring avian retrovirus. The transmission of ALV mainly occurs through the vertical route from the infected embryo to the offspring, and it can also be transmitted horizontally after direct or indirect contact with the infected chickens or the pollutants contaminated by the virus (4), which has a fast transmission speed and strong infectivity. Not only that, but ALV can also be transmitted through contaminated commercial live vaccines (5). Females infected with ALV-J through artificial insemination may transmit the virus vertically to their offspring through their eggs (6), so the only way to isolate the transmission of ALV is to eliminate positive chickens by testing for the pathogen (7). In response, there have been studies on the development of ALV vaccine (8), but the vaccine does not provide adequate protection for poultry. Therefore, early virus screening and rapid detection are essential for ALV prevention and control.

**Figure 1:**
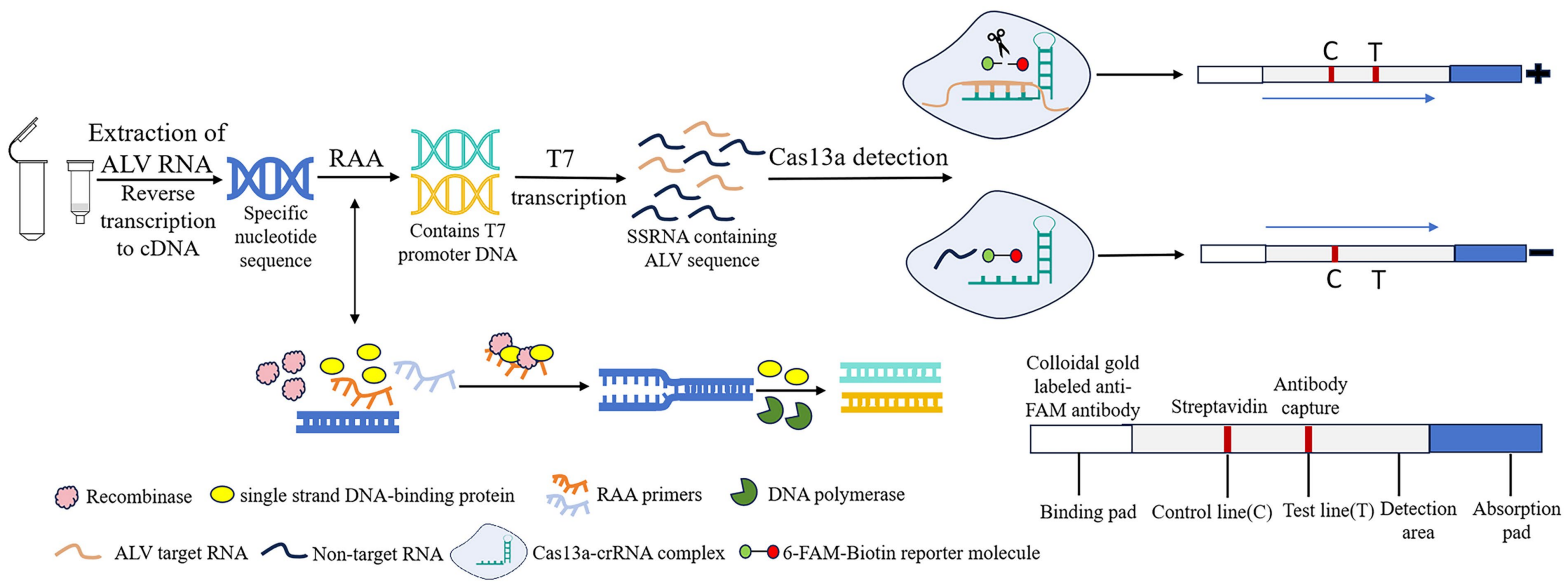
Schematic of RAA-CRISPR/Cas13a-LFD assay for ALV. RAA, recombinase-aided amplification; CRISPR, Clustered Regularly Interspaced Short Palindromic Repeats; Cas13a, CRISPR associated proteins 13a; LFD, lateral flow dipstick; ALV, Avian leukemia virus.

Recombinase Add Amplification (RAA) is a new type of rapid nucleic acid amplification technology that enables rapid expansion of DNA or RNA at a low temperature (usually 37°C), with the amplification product obtained in 30 min (9, 10). Clustered regularly interspaced short palindromic repeats (CRISPR) and its related proteins (Cas) constitute the CRISPR/Cas system. Its trans-cutting capability allows precise identification and cutting of specific nucleic acid targets. The system is used as a useful tool for genome editing and nucleic acid diagnostics due to its high sensitivity and specificity (11, 12). Among them, Cas13a has the ability to recognize RNA targets through CRISPR RNA (crRNA) and collateral cutting activity (13). Based on Cas13a protein, SHERLOCK system combines Cas13a with recombinase polymerase amplification (RPA) to achieve molecular diagnosis by utilizing collateral cleavage activity (14). In this experiment, the RAA-CRISPR/Cas13a-lateral flow dipstick (LFD) method was used to detect ALV, and the reaction principle was shown in Figure 1. After nucleic acid extraction of the pathogen to be tested, RAA amplification reaction is performed. The complex formed by recombinase, single-stranded binding protein and RAA primer scans double-stranded DNA and unrotated double-stranded DNA at the sequence homologous to the primer. The single-stranded binding protein prevents single-stranded DNA from refolding, and then DNA polymerase completes chain extension. After that, DNA containing T7 promoter is transcribed by T7 *in vitro* to form single-stranded RNA containing a large number of target sequences. Cas13a binds to crRNA to recognize the target sequence. If the complex recognizes the target sequence, the collateral cleavage activity of Cas13a nuclease is activated. 6-FAM-Biotin reported that the molecule was cut and bound to the colloidal gold labeled anti-FAM antibody complex, and the detection line (T) was positive. If the complex does not recognize the target sequence, the Cas13a nuclease is not activated, and the complex of the reporter molecule with colloidal gold labeled anti-FAM antibodies is captured by streptavidin, and only the control line (C) is negative. At present, RAA-CRISPR/Cas13a technology has been widely used in the detection of novel coronavirus (SARS-CoV-2) (15), plant viruses (16), African swine fever virus (ASFV) (17), and other fields.

In this study, RAA, CRISPR/Cas13a and LFD were combined to design RAA primer and crRNA according to the conserved sequence of ALV env gene. After establishing and optimizing the RAA-CRISPR/Cas13a-LFD reaction system, its specificity, sensitivity, repeatability and clinical reliability were evaluated, so as to establish a rapid, sensitive, specific and result-visualized ALV detection method, aiming to provide a powerful tool for the rapid detection, prevention and control of ALV in the field.

## Materials and methods

### Nucleic acid extraction

ALV, FAdV, IBDV, NDV, and MDV were all strains stored in the animal infectious disease laboratory of Hebei Agricultural University. IBV (AV1511) and ILTV (AV195) were purchased from the China Veterinary Drug Administration. The DNA of FAdV, ILTV, MDV, and RNA of ALV, IBDV, NDV, and IBV were extracted by TIANamp Virus DNA/RNA Kit (Tiangen Biochemical Technology Co., Ltd.), and the RNA were reverse transcribed into cDNA and stored at −20°C for future use.

### Primers and crRNA design and primer screening

**Figure 2:**
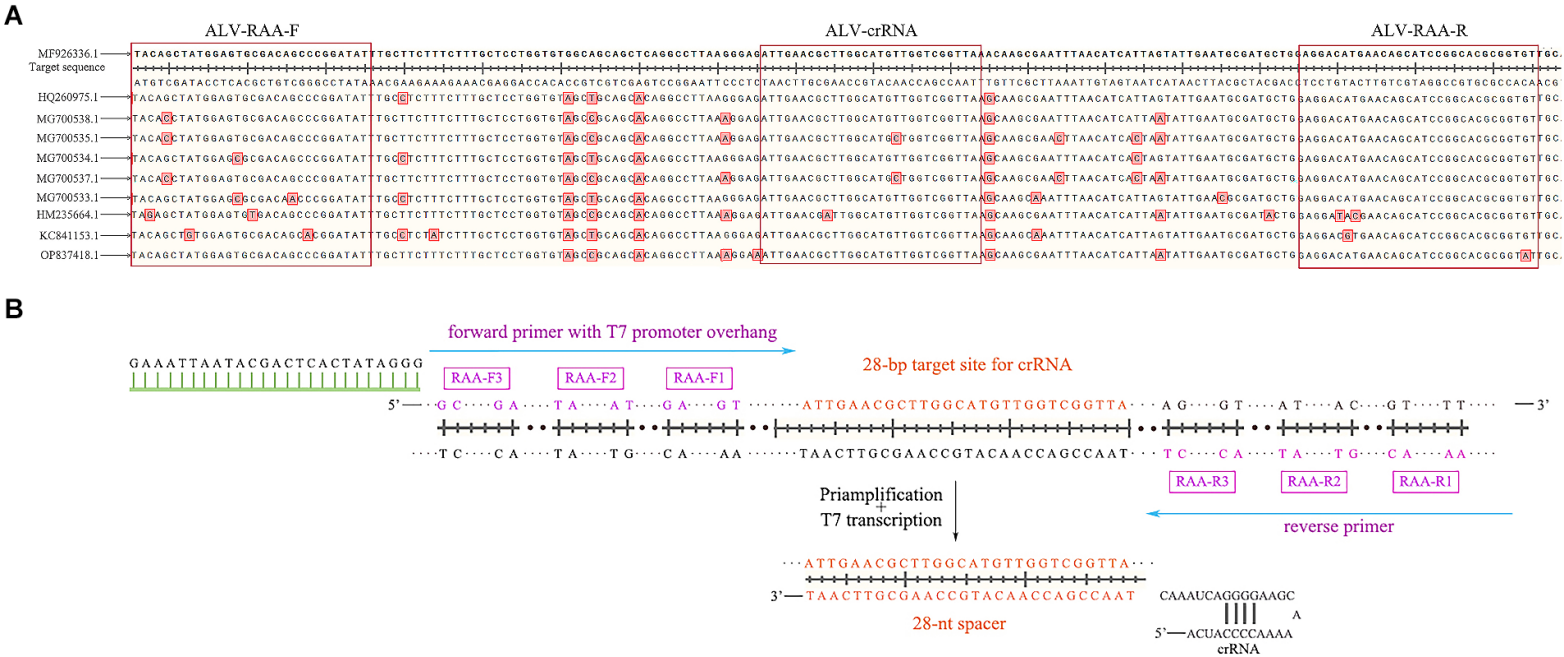
Schematic of primers and crRNA design. **(A)** The alignment results of gp37 sequences. **(B)** Schematic of primers and crRNA design. crRNA, CRISPR RNA.

According to the env gene sequence of ALV registered in GenBank (entry number: MF926336.1, OP837418.1, ON840108.1, HQ260975.1, MG700538.1, MG700535.1, MG700534.1, MG700537.1, MG700533.1, HM235664.1, KC841153.1), SnapGene and DNAMAN software were used for comparative analysis to determine the conserved sequence in the gp37 region of env gene (Figure 2A), and three pairs of specific RAA primers were designed (Table 1). Add on the forward primer 5’ end T7 RNA polymerase promoter sequences (T7; GAAATTAAT ACGACTCACTATAGGG; Figure 2B). Using ALV cDNA as template, RAA amplification reaction was performed. The reaction system was 50 μL, including buffer 25 μL, purified water 13.8 μL, forward primer 2.1 μL (10 μM), reverse primer 2.1 μL (10 μM), DNA template 2 μL, and 5 μL magnesium acetate. The amplification was performed at 37°C for 30 min, and then the amplified products were purified. The results were observed by 2% agarose electrophoresis, and a pair of optimal primers were selected. In addition, designing crRNA in the conserved sequence of the env gene, crRNA by LwaCas13a fixed stem ring structure (GTTTTAGTCCCCTTCGTTTTTGGGGTAGTCTAA ATCCCCTATAGTGAGTCGTATTAATTTC) and the length of target sequence of 28 nt (Table 1), which included in the primer amplification area. PCR-F/R was used to verify the sensitivity of PCR-agarose electrophoresis in this experiment. Both primer and crRNA were synthesized by Sangon Biotech (Shanghai) Company.

**Table 1:** Primer and crRNA sequences utilized in the study.

Name	Sequence (5′-3′)	Location (bp)
ALV-PCR-F	TACAGCTATGGAGTGCGACA	1,056–1,075
ALV-PCR-R	GTAAGAAATCGATGGCTGCT	1,246–1,265
ALV-RAA-F1	GATATTTGCTTCTTTCTTTGCTCCTGGTGT	1,081–1,110
ALV-RAA-F2	TACAGCTATGGAGTGCGACAGCCCGGATAT	1,056–1,085
ALV-RAA-F3	GCCGAGTCGTCTCTCGCCTGACTGCAGTGA	1,021–1,050
ALV-RAA-R1	AAGCAACACATCCCTTCCACGTCTTGACAC	1,285–1,314
ALV-RAA-R2	GTGTCCTTGCGCCAGGAGTAAGAAATCGAT	1,253–1,282
ALV-RAA-R3	ACACCGCGTGCCGGATGCTGTTCATGTCCT	1,206–1,235
T7-ALV-RAA-F2	GAAATTAATACGACTCACTATAGGGTACAGCTATGGAGTGCGACAGCCCGGATAT	1,056–1,085
ALV-crRNA	GAUUUAGACUACCCCAAAAACGAAGGGGACUAAAACUAACCGACCAACAUGCCAAGCGUUCAAU	1,136–1,163
T7 promoter	GAAATTAATACGACTCACTATAGGG	—

—, T7 promoter sequence; …, neck ring structure; ALV, Avian leukemia virus; crRNA, CRISPR RNA; RAA, recombinase-aided amplification; PCR, polymerase chain reaction.

### Construct standard plasmid

The cDNA of ALV was used as the template for PCR amplification. The PCR reaction system was 25 μL, including 2 × Taq Mix 12.5 μL, forward primer (10 μM) 0.5 μL, reverse primer (10 μM) 0.5 μL, template 1 μL, ddH_2_O 10.5 μL. The reaction procedure was as follows: 94°C 5 min, 94°C 30 s, 53°C 30 s, 72°C 30 s, 35 cycles, 72°C extended for 5 min. The PCR products were verified by 2% agarose gel electrophoresis. SanPrep Column DNA Gel Extraction Kit (Sangon Bioengineering Co., Ltd., Shanghai, China) was used for Gel recovery. The recovered DNA was connected to T-Vector pMD 19 vector (Takara Biomedical Technology Co., Ltd., Beijing, China) and introduced into DH5α Competent cells. After overnight culture, blue and white colonies were screened, white colonies were selected and plasmids were extracted with plasmid extraction kit (Tiangen Biochemical Technology Co., Ltd.). The standard plasmid was sequenced by Sangon Biotech.

According to the formula, the number of DNA copies contained in a plasmid per unit volume is calculated:


$$\begin{array}{l} \mathrm{Plasmid copy number}\;\left(\mathrm{copies/}\mu L\right)=[\mathrm{plasmid}\\ \mathrm{concentration}\;\left(\mathrm{g/}\mu L\right)\times 10^{-9}\times 6.02\times 10^{23}]\\ /\left\{\left[\mathrm{Vector length}\;\left(\mathrm{bp}\right)+\mathrm{fragment length}\;\left(\mathrm{bp}\right)\right]\times 660/\mathrm{gmol}\right\} \end{array}$$

The constructed standard plasmid was diluted to 10^9^–10^0^ copies/μL by 10-fold dilution method and stored at −20°C for later use.

### Establishment of RAA-CRISPR/Cas13a-LFD detection method

The RAA reaction was performed according to the instructions of the RAA nucleic acid amplification kit (Anhui Microanaly Genetech Co., Ltd., Hefei, China). The RAA reaction system was 50 μL, including buffer 25 μL, purified water 13.8 μL, forward primer 2.1 μL (10 μM), reverse primer 2.1 μL (10 μM), cDNA template 2 μL, and 5 μL magnesium acetate. Firstly, the reactants except magnesium acetate were successively added into the expansion tube containing lyophilization enzyme, and after gently mixing by hand, 5 μL of magnesium acetate was added to the lid, which was then amplified in a constant temperature amplification device at 37°C for 30 min.

RAA-CRISPR/Cas13a-LFD reaction system is 50 μL, Including 4 μL NTP buffer mix (New England Biolabs; 25 mmol/L), 2 μL RNase inhibitor (Takara Biomedical Technology Co., Ltd., Beijing, China; 40 U/μL), 4 μL Cas13a (Magigen China; 80 nmol/L), 2 μL crRNA (80 ng/uL), 2 μL RNA double-labeled probe (Bio-Lifesci, China; 100 μmol/L), 1 μL T7 RNA polymerase mix (New England Biolabs), 0.5 μL MgCl_2_ (Coolaber, China), 1 μL HEPES buffer (Solarbio, China), 28.5 μL enzyme-free sterile water and 5 μL RAA amplification product. The reactants were thoroughly mixed and incubated at 37°C for 25 min. Subsequently, 50 μL reaction products were added to LFD, and the results were observed within 3–5 min.

### Optimization of RAA-CRISPR/Cas13a-LFD reaction system

Factors affecting the amplification efficiency, including RAA amplification time, Cas13a concentration, crRNA concentration and CRISPR reaction time, were optimized by using the above reaction system. The RAA amplification time is set to start from 10 min and increase in gradient every 5 min. If clear detection lines appear, this time is selected as the optimal RAA amplification time. The concentration of Cas13a was set at 40 nmol/L and increased in a gradient of every 20 nmol/L, while other factors remained unchanged. When a clear detection line appeared, this concentration was selected as the optimal concentration of Cas13a. Under the optimal Cas13a concentration, the crRNA concentration was set to 10 ng/μL, and the gradient increase was every 10 ng/μL, and other factors remained unchanged. If a clear detection line appeared, the concentration was selected as the optimal crRNA concentration. Under the optimal concentration of Cas13a and crRNA, the CRISPR reaction time was set to start from 10 min and increase in gradient every 5 min until a clear detection line appeared.

### Specific detection

DNA of FAdV, ILTV and MDV and cDNA of ALV, IBDV, NDV, and IBV were used as templates, respectively, and ddH_2_O was set as negative control to evaluate the specificity of RAA-CRISPR/Cas13a-LFD assay.

### Sensitivity detection

The diluted ALV standard plasmid (10^7^–10^0^ copies/μL) was used as the template, and ddH_2_O was used as the negative control. PCR-Agar-gel electrophoresis, qPCR and RAA-CRISPR/Cas13a-LFD method were used for detection, and each method was repeated three times to evaluate its sensitivity.

The PCR reaction system was 25 μL, consisting of 2 × Taq Mix 12.5 μL, forward primer (10 μM) 0.5 μL, reverse primer (10 μM) 0.5 μL, template (10^7^–10^0^ copies/μL) 1 μL, ddH_2_O 10.5 μL. The reaction procedure was as follows: 94°C 5 min, 94°C 30 s, 53°C 30 s, 72°C 30 s, 35 cycles, 72°C extended for 5 min. The PCR products were subjected to agarose gel electrophoresis to determine the lowest detection limit.

The reaction system of qPCR was 20 μL, Including TB Green Premix Ex TaqTMII (TliRNaseH Plus; 2×; Takara Biomedical Technology Co., Ltd., Beijing, China) 12.5 μL, PCR forward and reverse primers (10 μM) 1.0 μL each, templates (10^6^–10^0^ copies/μL) 1.0 μL, ddH_2_O 9.5 μL, Reaction procedure: 95°C 30 s; The results were observed after 40 cycles at 95°C for 5 s and 60°C for 30 s.

The RAA-CRISPR/Cas13a-LFD assay used 10^5^–10^0^ copies/μL standard plasmid as a template and was carried out according to the optimized reaction system to determine the minimum detection limit of the method.

### Repeatability detection

Three ALV standard plasmids with different concentration after dilution (10^6^ copies/μL, 10^3^ copies/μL and 10^0^ copies/μL) were used as the template, and the test was repeated 3 times for each concentration, and ddH_2_O was set as the negative control. To evaluate the repeatability of RAA-CRISPR/Cas13a-LFD method.

### Clinical sample testing

The liver and kidney samples of 130 diseased chickens from northern China with preliminary clinical diagnosis of AL were detected by PCR-Agar-electrophoresis， RAA-CRISPR/Cas13a-LFD and qPCR (the samples were collected from 7 chicken farms). The coincidence rate of the RAA-CRISPR/Cas13a-LFD method and the two detection methods was calculated, and the feasibility of the application of the method in clinical samples was evaluated.

## Results

### Screening of RAA primers

**Figure 3:**
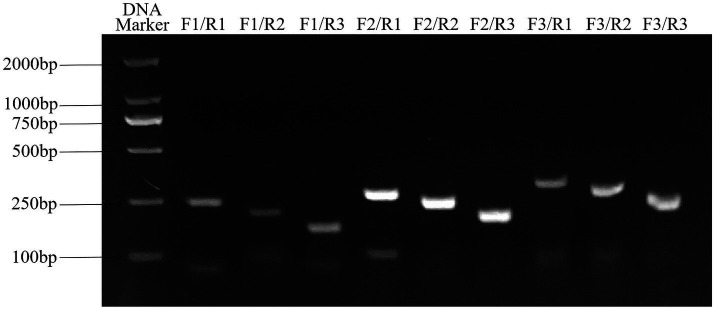
Primers screening for ALV detection by RAA assay. F2/R3 primer combination has good amplification efficiency and no non-specific amplification, which is the best primer combination. RAA, recombinase-aided amplification.

The results of primer screening showed that the amplification efficiency of RAA-F2/R1, F2/R2 and F2/R3 primer combinations was higher than that of other primer combinations, among which F2/R3 primer combination did not show non-specific amplification, so RAA-F2/R3 combination was selected as the best primer. The result is shown in Figure 3.

### Optimization of RAA-CRISPR/Cas13a-LFD reaction system

**Figure 4:**
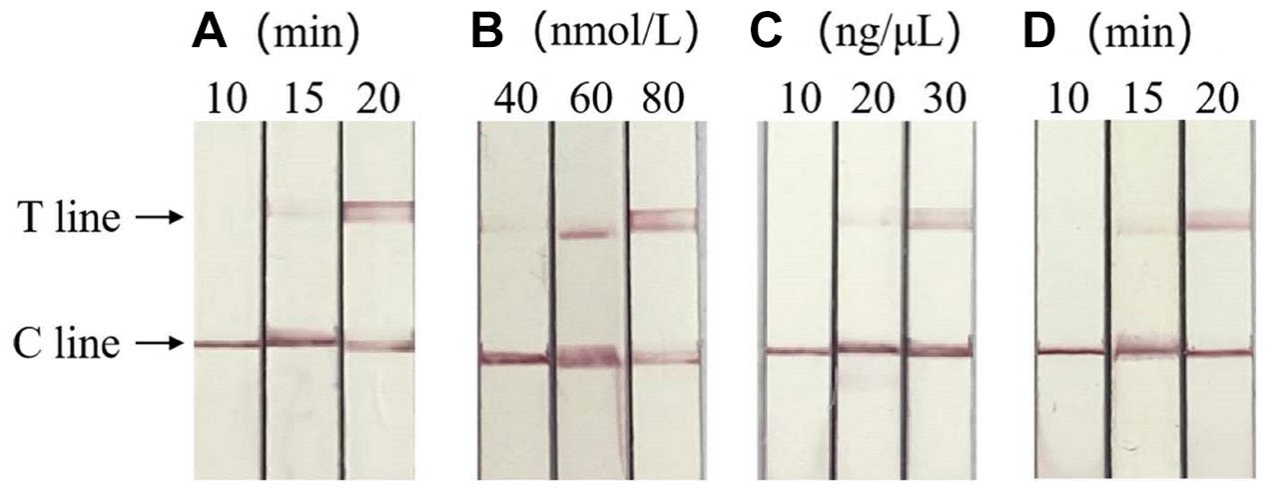
Optimization of RAA-CRISPR/Cas13a-LFD reaction system. **(A)** Optimization of RAA amplification time, the optimal RAA amplification time was 20 min. **(B)** Screening of the optimal concentration of Cas13a, the optimal Cas13a concentration was 60 nmol/L. **(C)** Optimum crRNA concentration screening, the optimal crRNA concentration was 30 ng/μL. **(D)** Optimum CRISPR response time screening, the optimal CRISPR reaction time was 20 min. C line, quality control line; T line, test line.

Based on the clear detection line of LFD, the optimal reaction conditions of RAA-CRISPR/Cas13a-LFD were verified as RAA amplification time of 20 min, crRNA concentration of 30 ng/μL, and CRISPR reaction time of 20 min, respectively. When the concentration of Cas13a is 60 and 80 nmol/L, clear detection lines appear in both cases, so the optimal concentration of Cas13a is 60 nmol/L. The result is shown in Figure 4.

### Specific detection

**Figure 5:**
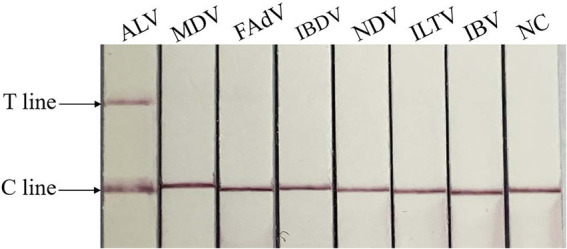
Specificity of RAA-CRISPR/Cas13a-LFD assay. NC, negative control; C line, quality control line; T line, test line. Only ALV showed T and C lines, while other viruses and negative control showed only C line.

The specificity test showed that only ALV was positive, and no test line was found between other viruses and negative controls, indicating that there was no cross-reaction between ALV and MDV, FAdV, IBDV, NDV, ILTV and IBV, and the method had good specificity. The result is shown in Figure 5.

### Sensitivity detection

**Figure 6:**
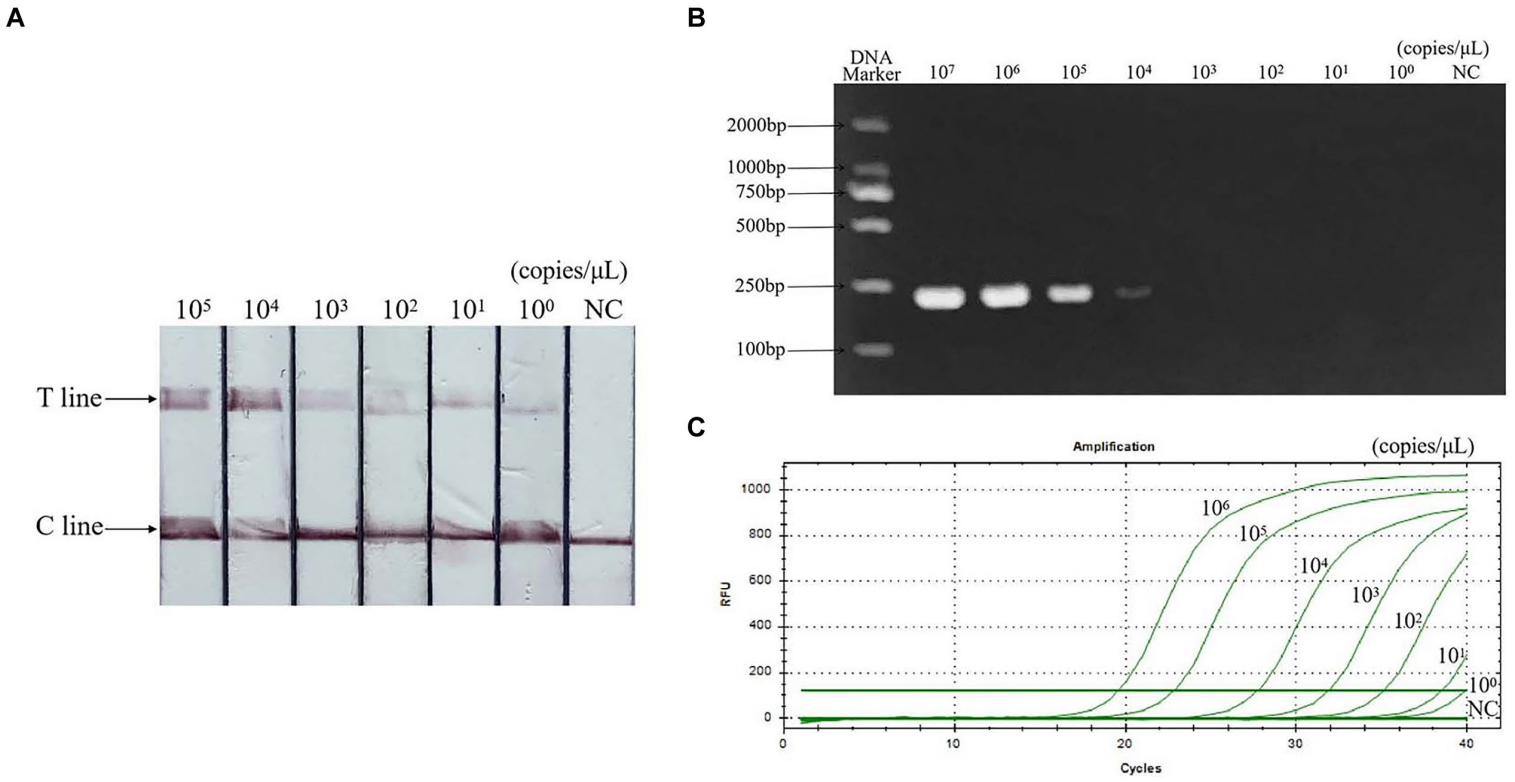
Sensitivity of RAA-CRISPR/Cas13a-LFD assay. **(A)** RAA-CRISPR/Cas13a-LFD method, the minimum detection limit is 10^0^ copies/μL. **(B)** PCR-agarose electrophoresis method, the minimum detection limit is 10^4^ copies/μL. **(C)** qPCR method, the minimum detection limit is 10^1^ copies/μL. NC, negative control; C line, quality control line; T line, test line.

Sensitivity evaluation results showed that the minimum detection limit of PCR was 10^4^ copies/μL, qPCR was 10^1^ copies/μL, and RAA-CRISPR/Cas13a-LFD was 10^0^ copies/μL. The sensitivity is 10 times that of qPCR and 10,000 times that of PCR. The result is shown in Figure 6.

### Repeatability detection

**Figure 7:**
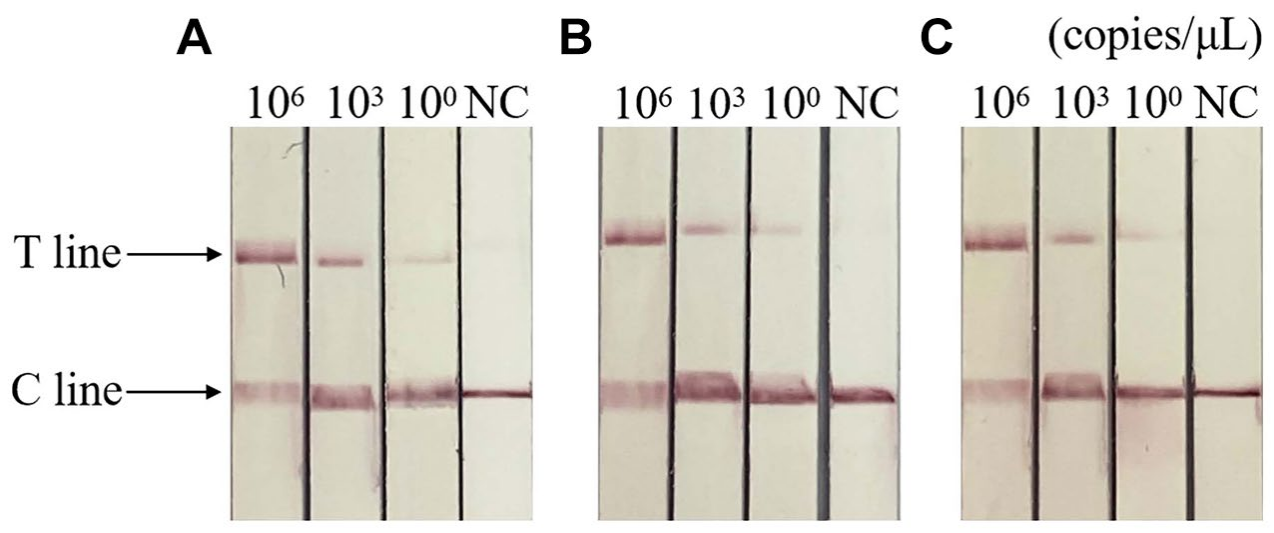
Repeatability of RAA-CRISPR/Cas13a-LFD assay. **(A–C)** Three repeated tests of three template concentrations. NC, negative control; C line, quality control line; T line, test line. T and C lines were shown in all three replication groups, and only C lines were shown in negative controls.

The results of repeatability test showed that the standard plasmids of different concentrations were positive, and the control group were negative, indicating that the detection method had good repeatability and stability. The result is shown in Figure 7.

### Clinical sample testing

ALV clinical sample test results showed (Table 2) that 42 positive samples were detected by RAA-CRISPR/Cas13a-LFD, and the coincidence rate with PCR-Agarose electrophoresis was 97.69% and 99.23% with qPCR method， indicating that RAA-CRISPR/Cas13a-LFD method has accurate detection ability and can be used for clinical detection.

**Table 2:** Clinical test results of the two methods.

Detection method	Positive (copy)	Negative (copy)	Positive rate (%)	Coincidence rate (%)
PCR-agarose electrophoresis	39	91	30.00	97.69
RAA-CRISPR/Cas13a-LFD	42	88	32.31	
				99.23
qPCR	41	89	31.54	

Coincidence rate (%) = (number of all positive samples + number of all negative samples in the two test methods compared)/total number of samples × 100%. PCR, polymerase chain reaction; RAA, recombinase-aided amplification; CRISPR, Clustered Regularly Interspaced Short Palindromic Repeats; Cas13a, CRISPR associated proteins 13a; LFD, lateral flow dipstick.

## Discussion

Since its first discovery in China in 1999, ALV-J has spread rapidly throughout the country. It was once a major disease endangering China’s poultry industry, with a mortality rate and morbidity as high as 50%, posing a great threat to the supply of laying chicken products for a long time and bringing serious economic losses to the poultry industry (18).

At present, the commonly used methods for detecting ALV in clinic include virus isolation, enzyme linked immunosorbent assay (ELISA), PCR, real-time fluorescence quantitative PCR (qPCR) and loop-mediated isothermal amplification (LAMP). Virus isolation and identification is the gold standard for detection of ALV, but it requires cell culture, which is time-consuming and laborious. LAMP detection method has high sensitivity, but its primer design is complex and easy to pollute (19, 20), which is not suitable for rapid detection at the grassroots level. ELISA method also needs to be combined with virus isolation and PCR, and the operation is complicated (21). Although the qPCR detection method has high sensitivity and specificity (22), and can be accurately judged, it requires complex and expensive instruments and operator expertise, and is not suitable for rapid on-site diagnosis. Therefore, establishing a rapid, accurate and convenient detection method is particularly important for the rapid diagnosis, prevention and control of AL. The CRISPR/Cas system is a diagnostic system first developed for molecular nucleic acids in 2016 (13). RAA can amplify viral nucleic acids at a temperature of 37°C–42°C without special temperature variable instruments, and can amplify the initial signal, which can be used to improve the sensitivity of the CRISPR/Cas system (12). With the rapid evolution of molecular diagnostics, the need for rapid tests has increased, and CRISPR/Cas systems, combined with various isothermal amplification technologies, have been used to detect various types of pathogens, including viral, bacterial and parasitic diseases. Zhang et al. (23) combined RAA with CRISPR-Cas13a system to detect avian influenza virus and used specific amplification of target gene fragments to provide a new detection method for avian influenza virus detection. An et al. (24) combined RPA with CRISPR-Cas13a system to provide a simple, rapid and specific detection method for Salmonella. Zhao et al. (25) combined RAA with CRISPR/Cas13a to establish a fast and sensitive rapid visual detection method for Toxoplasma gondii.

In this study, a RAA-CRISPR/Cas13a-LFD method was developed for the rapid detection of avian leukemia, and the results were determined by using transverse flow strips. The RAA-CRISPR/Cas13a-LFD method can complete the detection of target genes in only 40 min, and the targeted cutting ability of Cas13a binding with crRNA makes this method have strong specificity. The combination of RAA technology and CRISPR/Cas system improves the detection sensitivity. The minimum detection limit was 10^0^ copies/μL, which was 10 times more sensitive than qPCR and 10,000 times more sensitive than PCR. Miao et al. (26) used RAA-CRISPR/Cas13a-LFD to detect Nipah virus with a detection limit of 10^3^ copies/μL, which was slightly lower than the sensitivity of this study. Dou et al. (27) established a multiplex quantitative PCR method for the detection of avian leukemia in subgroups A, B, J and K, with a detection limit of 10^1^ copies/μL per response, but its operation is complex and the instrument is expensive. Xiang et al. (28) established a CPA method for the detection of avian leukemia subgroup J with a detection limit of 10^1^ copies/μL, but its response time was long and the sensitivity was only 10 times that of the PCR method. RAA-CRISPR/Cas13a-LFD method has good repeatability and stability, and high coincidence rate with PCR-Agarose electrophoresis method and qPCR method for the detection of the same batch of samples, which is suitable for clinical detection.

In this study, the RAA-CRISPR/Cas13a-LFD method for ALV field detection was established, which has the advantages of fast, sensitive and convenient, and provides a reliable technology for the diagnosis, prevention and control of AL.

## Data Availability

The datasets presented in this study can be found in online repositories. The names of the repository/repositories and accession number(s) can be found in the article/supplementary material.
